# Insights from Clinical Trials: Evidence-Based Recommendations for Induction Treatment of Newly Diagnosed Transplant-Eligible Multiple Myeloma

**DOI:** 10.3390/antib13040080

**Published:** 2024-09-29

**Authors:** Olga Lytvynova, Jenna Jwayyed, Daniel Pastel, Rohan Prasad, Jack Khouri, Louis Williams, Sandra Mazzoni, Shahzad Raza, Faiz Anwer

**Affiliations:** 1 Cleveland Clinic Akron General, Department of Internal Medicine, Akron, OH 44307, USA; 2 Department of Anesthesiology, Indiana University, Indianapolis, IN 46202, USA; 3 Cleveland Clinic Foundation, Taussig Cancer Center, Cleveland, OH 44106, USA

**Keywords:** multiple myeloma, transplantation, drug regimen, immunotherapy

## Abstract

Multiple myeloma (MM) is a hematological malignancy and poses significant therapeutic challenges. This review synthesizes evidence from pivotal clinical trials to guide induction treatment for transplant-eligible (TE), newly diagnosed MM (NDMM) patients. Emphasizing the evolution from three-drug to four-drug induction therapies, we highlight the integration of monoclonal antibodies, particularly CD38 recombinant monoclonal antibody agents, into treatment regimens. This analysis includes a comprehensive literature review of research from major databases and conferences conducted between 2010 and 2023, culminating in the detailed evaluation of 47 studies. The findings underscore the superiority of quadruple regimens in TE NDMM, notably those incorporating daratumumab, in achieving superior responses including progression-free survival (PFS), minimal residual disease (MRD) negativity, objective response rate (ORR), and overall survival (OS) when compared to triple-drug regimens. As treatment regimens evolve with additional agents, the improved outcomes with treatment-related adverse events should be carefully balanced. This review advocates for a paradigm shift towards quadruple induction therapies for TE NDMM, offers a detailed insight into the current landscape of MM treatment, and reinforces a new standard of care.

## 1. Introduction

Multiple myeloma (MM) is a B-cell malignancy derived from the uncontrolled proliferation of clonal plasma cells in the bone marrow, which produce monoclonal immunoglobulins. According to projections from the American Cancer Society (ACS), in the USA, an estimated 35,780 new cases of MM will be diagnosed in 2024, with a corresponding estimate of 12,540 deaths [1]. According to the revised International Myeloma Working Group (IMWG) criteria, the diagnosis of MM requires one of the myeloma defining events (MDE), which include hypercalcemia, renal failure, anemia, or osteolytic lesions in addition to either 10% or more clonal plasma cells on bone marrow examination or biopsy-proven plasmacytoma. Per the revised IMWG criteria, three biomarkers including clonal bone marrow plasma cells ≥ 60%, serum free light chains (FLC) ratio ≥ 100 (if involved light chains is ≥100 mg/L) and one or more one focal lesion of ≥5 mm on magnetic resonance imaging (MRI) without MDE is also classified as asymptomatic MM [2,3,4]. 

**Figure 1:**
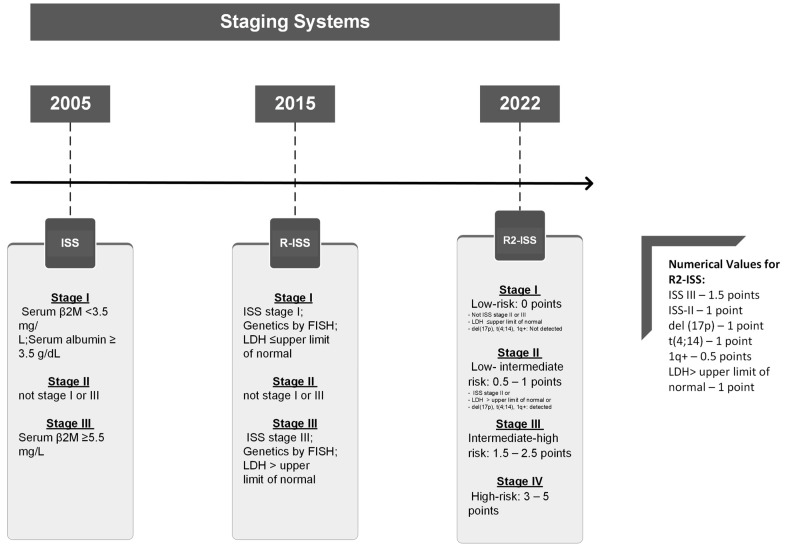
Multiple Myeloma staging evolution: a side-by-side comparison.

Risk stratification plays a pivotal role in prognosis and its use in tailoring treatment strategies for patients with MM may improve outcomes. Various genetic aberrations have been identified as high-risk prognostic indicators which adversely influence disease progression. Examples of adverse features include deletion of the short arm of chromosome 17 (del(17p)), t(4;14)), and t(14;16) duplication or amplification of 1q, all of which are associated with aggressive disease behavior. The staging of MM has evolved over the years, beginning with the International Staging System (ISS) in 2005, followed by the revised International Staging System (R-ISS) in 2015, which incorporated genetic mutations. The second revision of the International Staging System (R2-ISS) improved the prognostic assessment in the previously classified intermediate risk category of patients (Figure 1) [2,5,6,7,8,9].

Survival in MM has improved significantly in the last 15 years with the incorporation of novel agents. Initially, immunomodulatory agents (IMiDs) (thalidomide, lenalidomide) and proteasome inhibitors (PI) (bortezomib) have shown remarkable activity against MM. In the last decade, incorporation of third generation of IMiDs (pomalidomide), additional PI (carfilzomib, ixazomib), monoclonal antibodies against CD38 (daratumumab, isatuximab), a SLAMF7-directed immunostimulatory antibody (elotuzumab), an exportin 1 inhibitor (selinexor), two CAR-T cell therapies against B-cell maturation antigen (BCMA) (ida-cel, cilta-cel), anti-BCMA bispecific antibodies (Teclistamab, Elranatamab) and a G-protein-coupled receptor, Family C, Group 5, Member D (GPRC5D) bispecific antibody (talquetamab) have shown promising efficacy [10,11,12,13]. Despite these advances, MM is still incurable. Newly diagnosed MM (NDMM) is generally treated with combinations of three or four drugs which include IMiD, PI, anti-CD38 monoclonal antibody and dexamethasone for 4–6 cycles followed by autologous stem cell transplantation (ASCT) [14]. 

This review primarily focuses on the identification and assessment of the efficacy and toxicity of available induction treatment options for NDMM in transplant-eligible adults. Particular emphasis is placed on exploring the role of anti-CD38 monoclonal antibodies, or other quadruple therapy options compared to standard triple therapy approaches. Most studies used PFS, ORR, and CR as their primary endpoints as a way to determine treatment efficacy, although several studies used other endpoints. By thoroughly analyzing the efficacy and safety of these quadruple therapies, we aim to contribute to the understanding of the evolving landscape of multiple myeloma therapies, paving the way for enhanced patient outcomes and ultimately improved quality of life.

## 2. Materials and Methods

**Figure 2:**
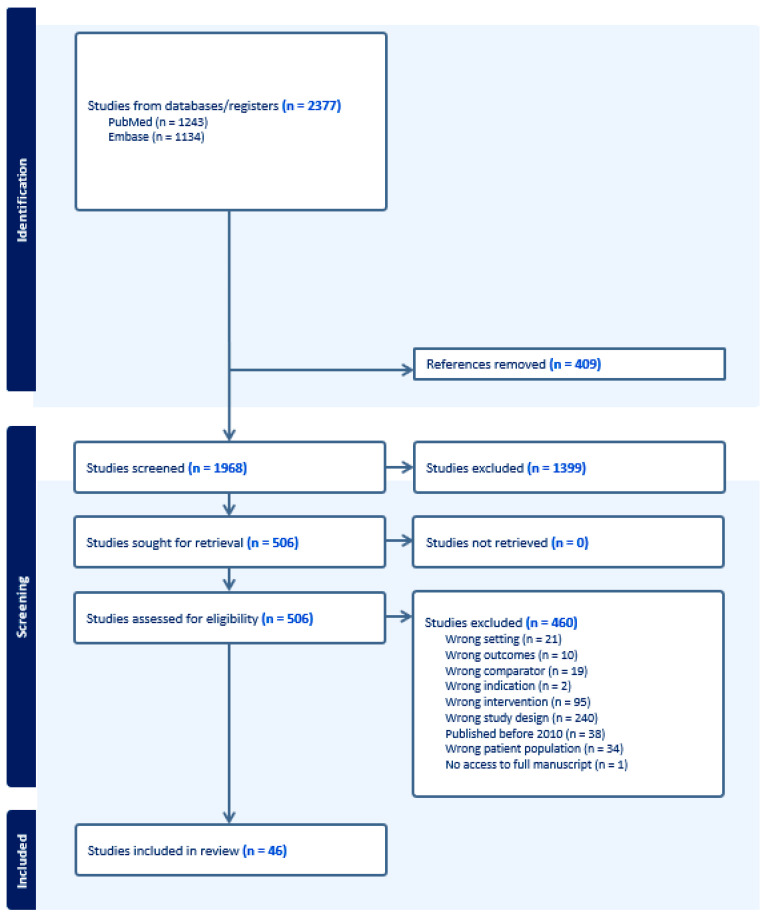
PRISMA flowchart of included articles.

To comprehensively assess the contemporary literature on treatment options for transplant-eligible newly diagnosed multiple myeloma (TE-NDMM), an extensive literature search was conducted. This search aimed to include all Phase II and Phase III studies published in English language by utilizing specific search terms covering the period from 1 January 2010 to 31 December 2023. The databases employed for this search were PubMed and Embase, a total of 2377 publications were initially retrieved. These studies were then imported into the systematic review software, Covidence [15] as depicted in Figure 2. After the initial screening phase involving the review of titles and abstracts of 1968 citations, 1399 were determined to be irrelevant to the research objectives and were subsequently excluded. 

Following the removal of duplicates, 506 citations advanced to the full-text review phase. At this stage of screening 460 studies were excluded due to a variety of reasons including inappropriate study design, intervention type, publication date (i.e., before 2010), patient demographic, study setting, comparator, outcomes, indication, or incomplete information about outcomes. Consequently, 46 studies met the inclusion criteria and were selected for detailed analysis. Moreover, this review was augmented by incorporating the most recent findings presented at the American Society of Hematology (ASH) conference held in December of 2023. 

## 3. Results

### 3.1. Insights from Quadruple Therapy Clinical Trials 

#### 3.1.1. Daratumumab-Containing Induction Therapy Trials 

This comprehensive data from the trials are summarized in Table 1 and Table 2.

- Perseus Trial (Phase III)—Dara-VRd vs. VRd:
  This landmark trial enrolled 709 patients to evaluate subcutaneous daratumumab in combination with bortezomib, lenalidomide, and dexamethasone (Dara-VRd) during the induction/consolidation phase and Dara-R for maintenance versus, VRd for induction/consolidation followed by lenalidomide alone for maintenance. The primary endpoint was PFS and at a median follow-up of 47.5 months, the PFS rates were significantly improved in the Dara-VRd group compared to the VRd group (84.3% vs. 67.7%, *p* ≤ 0.001). The ORR was not reported in the study, but the CR rate was higher in the Dara-VRd group than in the VRd group (87.9% vs. 70.1%, *p* ≤ 0.001). The benefit was noted across all subgroups, including high-risk patients. The most common Grade 3 and 4 treatment-emergent adverse events (TEAEs) for the Dara-VRd arm vs. VRd alone were neutropenia, thrombocytopenia, and diarrhea. Serious TEAEs leading to treatment discontinuations were noted to be higher in the Dara-VRd group versus the VRd group (57% vs. 49.9%, respectively) [15,16].

- Griffin Trial (Phase II)—Dara-VRd vs. VRd:
  In this study, 207 patients were randomized to receive either Dara-VRd or VRd therapy for four cycles of induction, followed by ASCT, then two consolidation cycles, and finally maintenance therapy for up to two years with Dara-R vs. R alone. Stringent complete response (sCR) post-ASCT consolidation therapy was the primary endpoint with a median follow-up of 13.5 months and it showed better sCR rates with Dara-VRd vs. VRd (42.4% vs. 32%, *p* = 0.068). The final analysis after a median follow-up of 49.6 months showed the PFS was higher in the Dara-VRd group compared to the VRd alone group (87.2% vs. 70%, *p* = 0.032). There is a trend toward improved PFS Dara-VRd vs. VRd (hazard ratio (HR) (95% CI)) in patients > or = 65 years (0.29 (0.06–1.48)), with high risk cytogenic abnormalities (HRCAs) (0.38 (0.14–1.01)), and with gain/amp (1q21) (0.42 (0.14–1.27)). The ORR was measured at the 13.5-month follow-up and was higher in the Dara-VRd than the VRd alone groups (99% vs. 91.8% *p* ≤ 0.016). Most common grade 3 and 4 adverse events in both arms included neutropenia, lymphopenia, and thrombocytopenia. Treatment discontinuation due to TEAEs occurred in fewer patients in the Dara-VRd group than the VRd alone group (15.2% vs. 20.6%, respectively) [17,18,19,20,21].

- Cassiopeia Trial (Phase III)—Dara-VTd vs. VTd:
  This two-part study compared daratumumab, bortezomib, thalidomide, and dexamethasone (Dara-VTd) versus VTd alone for the induction and consolidation cycles for 1085 patients who received ASCT, following this treatment. In Part 2, patients were randomly assigned to receive daratumumab maintenance every 8 weeks for up to 2 years vs. observation. In part one, the primary endpoint was sCR 100 days after transplantation and showed that in the Dara-VTd group had higher sCR than the VTd group (29% vs. 20%, *p* ≤ 0.001), median PFS was not achieved. The primary end point for Part 2 was PFS based on the second randomization and showed significant benefit in the VTd plus daratumumab group when compared to the VTd with only observation; no significant difference in PFS was seen in the original group receiving daratumumab for induction. The ORR of 92.6% was noted in the Dara-VTd group compared to the 89.9% VTd group. Subgroup analysis of 15.5% patients with high risk cytogenic abnormality analyzed sCR as a primary endpoint. sCR rates were higher within Dara-VTd vs. VTd group (28.9% vs. 20.3%, respectively), (95% [1.21–2.12]). During Part 2 of the study, the rate of Grade 3 or 4 adverse events was 28% in the daratumumab group vs. 24% in the observation group. Serious adverse events occurred in 23% vs. 19% of patients, respectively [22,23,24].

- Master Trial (Phase II)—Dara-KRd:
  This study assessed the combination of daratumumab, carfilzomib, lenalidomide, and dexamethasone (Dara-KRd) in 123 MM patients. The next generation sequencing (NGS) based minimal residual disease (MRD) (<10–5) status was used for the assessment of treatment cessation based on 2 consecutive negative MRD results. MRD was evaluated at the end of induction, post ASCT, and every 4 cycles of consolidation. The primary endpoint was achieving MRD negativity at any point during the study with 38% reaching MRD post-induction, 65% reaching MRD post-ASCT and 80% reaching MRD-directed consolidation (78%, 82% and 79% of patients with 0, 1 and 2+ HRCA, respectively); 2-year PFS was 87% for the MRD 10–5 cohort (91%, 97%, and 58% for patients with 0, 1, and 2+ HRCA, respectively) compared to 81% for the MRD 10–6 cohort. The ORR was 95% at 8 months in the study. The study reported that all 123 participants experienced at least one treatment emergent adverse event, 74% experienced grade 3–5 adverse events. Two patients discontinued carfilzomib due to AE and two patients discontinued lenalidomide due to AE. There were three treatment-related deaths reported [25].

- Lyra Trial (Phase II):—Dara-Vcd:
  The study evaluated a combination treatment regimen consisting of daratumumab, bortezomib, cyclophosphamide, and dexamethasone (Dara-Vcd) in 87 patients with NDMM and 14 patients with relapsed MM with a specific focus on achieving MRD negativity and understanding the regimen’s safety profile. The primary endpoint was very good partial response or better (VGPR+) following 4 cycles of induction therapy. In the NDMM cohort the rate of CR+VGPR after 4 cycles of induction was 44.2% and the ORR was 79.1%. At the end of the induction therapy, CR+VGPR was 55.8% and the ORR was 81.4%. Median PFS was not reached among those with standard risk and was 33.1 months among those with high risk, while the 36-months PFS was 87.5%for standard risk patients vs. 45.2% for high risk patients. All participants experienced at least one TEAEs. The most common AE included fatigue and neutropenia, which were the most frequent Grade 3–4 TEAEs. Infusion reactions occurred in 54% of patients, primarily during the first dose, and were predominantly mild, with only 2% being Grade 3. Three treatment-related deaths were reported in the study [26,27,28].

#### 3.1.2. Isatuximab-Containing Induction Therapy Trials

This comprehensive data from the trials is summarized in Table 3 and Table 4.

- GMMG-HD7 (Phase III)—Isa-VRd vs. VRd:
  The trial involved a cohort of 660 patients divided into the isatuximab, bortezomib, lenalidomide, and dexamethasone (Isa-VRd) arm versus VRd arm for induction therapy. The primary endpoint was MRD negativity assessed by next generation flow (NGF) after induction and resulted in 50.1% vs. 35.6%, *p* < 0.001, respectively. The subgroup analysis evaluated patients with the high-risk cytogenetics (45.2% of patients) and the ultra-high-risk cytogenetics (14.1% of patients). In patients with high-risk cytogenetics, MRD negativity resulted in 50.4% in Isa-VRd arm vs. 37.4% VRd arm (95% CI, 1.04–2.79). In patients with ultra-high-risk cytogenetics, MRD negativity resulted in 56.3% vs. 44.1% with Isa-VRd arm vs. VRd arm, respectively (95% CI, 0.67–3.99). Neither PFS nor ORR were secondary endpoints in this study so were not reported. At least one Grade 3 or higher AEs were reported in 63% of Isa-VRd group vs. 61% of VRd group. Five treatment-related deaths were reported: one death in the Isa-VRd vs. four deaths in the control group [29,30,31,32].

#### 3.1.3. Carfilzomib-Containing Induction Therapy Trials

- Myeloma XI+ Trial (Phase III)—KRdc vs. Rdc/Tdc:
  This study compared carfilzomib, lenalidomide, dexamethasone, and cyclophosphamide (KRdc) to a regimen of dexamethasone and cyclophosphamide with either lenalidomide or thalidomide (Rdc/Tdc) in 1056 patients. Patients underwent genetic profiling and were stratified into no-hit (standard risk), single-hit (high risk), or double-hit (ultra high risk). The primary endpoints in the study were PFS and OS. After a median follow-up of 34.5 months, the KRdc group showed significantly improved PFS compared to those receiving the control regimens (Rdc/Tdc). Specifically, KRdc patients were more likely to achieve at least VGPR by the end of induction therapy, with an 82.3% response rate compared to 58.9% in the control group. Additionally, the study noted that a higher percentage of KRdc patients achieved MRD negativity. Stratifying patients by genetic profile, lenalidomide maintenance had the biggest effect on PFS in patients with single hit (HR 0.38; 95% CI, 0.25–0.58; *p* < 0.0001), followed by patients patients with no-hit (HR, 0.46; 95% CI, 0.33–0.64; *p* < 0.0001) and those with double-hit (HR, 0.55; 95% CI, 0.34–0.90; *p* = 0.17). The incidence of serious AEs was higher in the KRdc group (69.5%) compared to the control groups (55.3%) [33,34].

#### 3.1.4. Elotuzumab-Containing Induction Therapy Trials

- SWOG-1211 Trial (Phase II)—Elo-VRd vs. VRd:
  This study compared the incorporation of elotuzumab into the bortezomib, lenalidomide, and dexamethasone (Elo-VRd) induction regimen for 100 high-risk multiple myeloma patients followed by attenuated maintenance dose with the same regimen. The primary endpoint analyzed was PFS at the time of disease progression and found that the PFS of the Elo-VRd group was 65% versus 60% in the VRd group, this finding was not found to be significant. The median time to follow-up was 53 months. The ORRs between the two groups were 83% and 88% for the Elo-VRd and VRd groups, respectively; this was also not significant (*p* = 0.29). There were no significant differences in Grade 3–5 infections between the Elo-VRd and VRd groups. However, there was one treatment-related death reported in the Elo-VRd group [35].

- EVOLUTION (Phase II)—VdcR-, VRd-, Vdc-, and Vdc-modified:
  This multiphased trial examined the tolerability of bortezomib, cyclophosphamide, lenalidomide, and dexamethasone (VdcR) and to study the combination concurrently with VRd and Vdc.
  The primary objective was to determine the combined rate of CR plus VGPR for the VdcR, VRd, and Vdc regimens. Secondary objectives included safety and tolerability, ORR, time to response, time to progression (TTP), PFS, and OS. The 140 patients were enrolled, including 7 in the VdcR arm treated at MTD in Phase 1. The median follow-up was 20 months (range: 0–30); 20, 20, 22, and 15 months, respectively, for the VdcR, VRd, Vdc, and Vdc-mod arms. Overall, 36%, 41%, and 23% of patients, respectively, had ISS Stage I, II, or III disease. High-risk MM was present in 17% of patients as determined by cytogenetics.
  Following the completion of four cycles, the ORR was 80% in VdcR, 73% in VRd, 63% in Vdc, and 82% in Vdc-modified arms, while patients achieving VGPR or better were 33%, 32%, 13%, and 41%, in the arms, respectively. Across all treatment cycles, VGPR or better was achieved in 58% in VdcR, 51% in VRd, 41% in Vdc, and 53% in Vdc-modified. Across all cycles, the ORR was 88%, 85%, 75%, and 100%, respectively. The median time to best response was 105 days in the VdcR, 91 days in the VRd, 118 days in Vdc, and 85 days in the Vdc-modified arms. At least one grade ≥ 3 AE was seen in ∼80% of patients in each arm. AEs leading to discontinuation were seen in 21%, 19%, 12%, and 6% in the VdcR-, VRd-, Vdc-, and Vdc-modified arms, respectively [36].

### 3.2. Insights from Triple Therapy Clinical Trials

#### Summary of Individual Triple Therapy Trials

These trials are collectively highlighted in Table 5 and Table 6.

- PETHEMA/GEM2012 (Phase III)—VRd:
  This trial of 458 patients with NDMM underwent six cycles of bortezomib, lenalidomide, and dexamethasone (VRd), followed by ASCT, then conditioned with busulfan and melphalan versus melphalan and consolidated with 2 cycles of VRd. The primary endpoint was PFS with a median follow-up time of 22.4 months, the median PFS was not reached in either arm. The ORR was not analyzed in this study, but the VGPR was noted to be improved with subsequent cycles from 55.6% by cycle 3 to 70.4% after induction (cycle 6). The 34.8% of the total population had high risk cytogenetics. The 81.5% of high-risk cytogenetic patients achieved partial response or better. The Grade >= 3 adverse events during the induction phase was 3.9% and 3.1% had >=1 TEAE leading to discontinuation of induction. In addition, 2% of patients died during the induction phase [38].

- ENDURANCE (Phase III)—VRd vs. KRd:
  In this study, 1087 patients with NDMM were randomized into two groups that both received induction with lenalidomide and dexamethasone and one group each receiving either bortezomib or carfilzomib (VRd vs. KRd). The primary endpoint was PFS with a median follow-up of 9.4 months. The median PFS in the VRd group was 37 months and 28 months in the KRd group. The ORR was 84% of VRd and 87% of KRd patients (*p* < 0.01). The 11% of patients were identified to have high risk cytogenetic features with MRD negativity reported 21% for the VRd cohort and 29% for the KRd cohort (*p* = 0.30). Serious AE were recorded as 22% vs. 45% in the KRd and VRd groups, respectively. In the VRd group, there were <1% treatment-related deaths, and in the KRd group, there were 2% [39].

- Reeder Trial (Phase II)—Vcd:
  This study of 63 patients evaluated bortezomib, cyclophosphamide, and dexamethasone (Vcd) in bortezomib with dexamethasone (Cohort 1 with standard dosing) vs. high-dose bortezomib with 2 cycles of high-dose dexamethasone followed by 2 cycles of low dose dexamethasone (Cohort 2 with modified dosing). The primary endpoint was the response following completion of the 4 cycles which was 89% for all patients. The PFS at five years was 42%, and the median follow-up was 12.4 months. In Cohort 1, the ORR was 96% vs. 92% in Cohort 2. The 38% of total patients were considered high risk. The high risk cohort in comparison to standard risk cohort demonstrated lower 5 year PFS (33% vs. 48%) and lower OS (54% vs. 81%). Grade 3 AEs were 48% in Cohort 1 and 37% in Cohort 2, while Grade 4 AEs were 12% in Cohort 1 and 3% in Cohort 2 [40,41,42].

- NCT02405364 (Phase II)—KRd:
  This study of 48 patients investigated a triple therapy regimen that consisted of carfilzomib, lenalidomide, and dexamethasone (KRd) in NDMM patients. The primary endpoint was sCR at the completion of consolidation which was reached by 61.9% of patients and was 56.5% (*p* = 0.0172). The median follow-up was 60.5 months with a 5-year PFS of 45.1%. The 20% of patients were considered high-risk, with 78% of them achieving at least CR at completion of consolidation and undetectable MRD. Serious AEs were reported in 8.7% of patients, but no treatment-related deaths occurred. At least 1 TEAE was noted in 97.8% of patients [43].

- FORTE Trial (Phase II)—KRd vs. KCd:
  This study enrolled 477 patients and was divided into three cohorts: KRd plus ASCT, KRd for 12 cycles, and KCd plus ASCT. The 356 patients who were eligible for maintenance treatment were subsequently randomized into two cohorts: KR vs. R monotherapy. The median follow-up from the first randomization was 50.9 months, and from the second randomization, it was 37.3 months. The primary endpoints were VGPR—which was achieved by 70% of patients receiving KRd compared to 53% of patients receiving KCd—and PFS. The 4-year PFS in patients with MRD negativity was 87% in the KRd-ASCT cohort, 92% in the KRd12 cohort, and 76% in the KCd-ASCT cohort. The 3-year PFS from the second randomization was 75% in the KR cohort compared to 65% in the R monotherapy cohort. One-year MRD negativity between patients with zero HRCA and one HRCA was 35% vs. 41% respectively. Risk of progression or death in patients with one HRCA vs. zero HRCA was higher (HR 1.68; 95% CI, 1.01–2.80, *p* = 0.048). TRAEs occurred in 11% of the KRd-ASCT group, 19% of the KRd12 group, and 11% of the KCd-ASCT group [44,45,46].

- DETERMINATION Trial (Phase III)—VRd vs. VRd+ASCT:
  This study consisted of 722 patients who were randomly assigned to receive 3 cycles of bortezomib, lenaliodmide, and dexamethasone (VRd), stem cell mobilization, and then 5 more VRd cycles (Arm A) or melphalan + ASCT and 2 VRd cycles (Arm B). Both arms received R maintenance until progression or intolerance. The primary endpoint was PFS. Patients were randomly assigned to Arms A and B, respectively; 14% and 13% had ISS stage III MM, and 18% each had high-risk cytogenetics [t(4;14), t(14;16), del17p]. In the respective arms, 291 and 290 pts received R maintenance for a median duration of 36 and 41 months. After median follow-up of 76 months, median PFS was 46.2 vs. 67.6 months in Arm A vs. B (HR 1.53; 95% CI, 1.23–1.91; *p* < 0.0001). With 90 vs. 88 patients having died in Arm A vs. B. A 4-year OS was 84% (95% CI, 80–88%) vs. 85% (95% CI, 81–88%); HR 1.10 (95% CI, 0.81–1.47; *p* = 0.274). Grade ≥ 3 related adverse events were less common in Arm A vs. B (78% vs. 94%; hematologic: 61% vs. 90%, *p* < 0.0001); 10% vs. 11% had secondary malignancies (ALL, 7 vs. 3 patients, *p* = 0.22; AML/MDS, 0 vs. 10 patients, *p* = 0.002) [47,48].

- IFM 2009 Trial (Phase III)—VRd vs. VRd+ASCT:
  This study treated 700 patients with MM with induction therapy of three cycles of bortezomib, lenalidomide, and dexamethasone (VRd). Followed by consolidation therapy in 2 randomized groups of 350 patients. The first receiving five additional cycles of VRd and the second group with high-dose melphalan plus stem cell transplantation followed by two additional cycles of VRd. Patients in both groups received maintenance therapy with lenalidomide for 1 year. The primary end point was PFS, which was significantly longer in the transplant group than in the group that received VRd alone (50 months vs. 36 months, respectively; adjusted HR for disease progression or death, 0.65; *p* < 0.001). OS at 4 years did not differ significantly between the transplant group and the VRd-alone group (81% and 82%, respectively). The rate of Grade 3 or 4 neutropenia was significantly higher in the transplant group than in the VRd-alone group (92% vs. 47%), as were the rates of grade 3/4 gastrointestinal disorders (28% vs. 7%) and infections (20% vs. 9%) [49].

- SWOG SO777 Trial (Phase III)—VRd vs. Rd:
  This trial randomly assigned 525 patients to receive either an initial treatment of bortezomib, lenalidomide, and dexamethasone (VRd group) or lenalidomide and dexamethasone alone (Rd group). The VRd regimen was given as 8 21-day cycles. The Rd regimen was given as 6 28-day cycles. The primary endpoint was PFS. Median PFS was significantly improved in the VRd group (43 months vs. 30 months in the Rd group; stratified HR 0.712, 96% CI, 0.56–0.906; one-sided *p* = 0.0018). Adverse events of Grade 3 or higher were reported in 82% in the VRd group and 75% in the Rd group; 23% and 10% patients discontinued induction treatment because of adverse events, respectively [50].

- GIMEMA-MMY-3006 Trial (Phase III)—VTd vs. Td:
  This was a trial of 480 patients with a median follow-up of 124.1 months and showed a 10-year PFS of 34% (95%, Cl 28–41) for VTd compared with a 17% with Td (Cl 13–23); for the Td group hazard ratio 0.62 (95% Cl 0.50–0.77; *p* < 0.0001). The 60% (95%, Cl 54–67) of patients in the VTd group were alive at 10 years vs. 46% (Cl 40–54) of patients in the Td group (HR 0.68; 95%, Cl 0.51–0.90; *p* = 0.0068). Incorporation of VTd into double autologous HSCT resulted in clinically meaningful improvements in long-term PFS and OS [51,52].

- Carthadex Trial (Phase II)—KTd:
  The trial investigated 111 patients with dose escalation of carfilzomib in the KTd regimen and showed a median PFS of 58 months with a median follow-up of 58.7 months. When eight cycles of KTd were completed, it resulted in only slightly higher percentages of CR and VGPR as compared to when only four cycles were completed. However, more cardiac events and premature treatment discontinuation were observed when eight cycles were completed. When four cycles of KTd were completed, almost all patients achieved at least a PR. Moreover, response percentages after ASCT, as well as after consolidation, were comparable between the four- and eight-cycle groups and more importantly, PFS and OS were not different [53].

## 4. Discussion

Recent MM trials have focused on comparing the efficacy and safety of four drug regimens compared with three-drug regimens in TE-NDMM patients. A comprehensive analysis of these trials was performed, in particular, we analyzed data for Perseus (Dara-VRd vs. VRd), GRIFFIN trial (Dara-VRd vs. VRd), CASSIOPEIA (Dara VTd vs. VTd), Master trial (Dara-KRd), Lyra trial (Dara-CyBorD), GMMG-HD7 (Isa-VRd vs. VR), Myeloma XI+ (KRdc vs. Rdc/Tdc), SWOG-1211 (Elo-VRd vs. VRd), and Ludwig’s trial (VTdc vs. VTd). These trials shed light on the remarkable advantages associated with quadruple regimens [15−36]. Quadruple regimens, especially with CD38 targeting antibodies, have consistently demonstrated improved long-term outcomes with superior PFS. Despite the addition of these agents, many quadruple regimens have maintained manageable toxicity profiles, allowing for sustained treatment delivery, and leading the way for a paradigm shift in the management of TE NDMM. These findings necessitate a reconsideration of therapeutic strategies to incorporate CD38-targeting-antibody-based four-drug regimens into routine practice. Using four different drug regimens may lead to distinct toxicity profiles and higher initial costs. For example, daratumumab has been increasingly used as an agent and has been particularly associated with increased cost. The estimated cost for a standard 23-course treatment of daratumumab is approximately USD 162,332 when calculated for a 16mg/kg dosing regimen for an average 78 kg individual [54]. However, these regimens often result in longer-lasting and deeper remissions, which can reduce the need for ongoing treatments and single-drug maintenance therapies, ultimately improving patient responses. Additionally, patients with sustained MRD-negative results and standard genetics may be eligible to discontinue treatment altogether.

In April 2024, FDA Oncologic Drugs Advisory Committee recognized MRD negative (×105) as a surrogate marker of longer PFS (global odds ratio 4.72), superior OS (global odds ratio 4.02) as an intermediate clinical endpoint, measured at 9–12 months (+/− 3 months) for accelerated drug approvals for the treatment of Myeloma [55]. The Master trial demonstrated that 80% of patients achieved MRD negativity, and the Cassiopeia trial resulted in higher MRD negativity (63.7% vs. 43.5%) in the Dara-VTd arm. The Griffin Phase II study compared daratumumab-VRd vs. VRd; it showed a striking difference in 48-month PFS favoring the daratumumab-VRd arm, 87.2% compared to 70% in the control group. These landmark trials, with higher sustained MRD negative rates, support the transition towards quadruple therapies as the new standard of care for TE NDMM patients. Despite MRD negative status, PFS is adversely impacted by high-risk genetics, in the Master trial, 71% of patients with sustained MRD negative status (MRD-SURe), the 2-year cumulative risk of progression was 9%, 9%, and 47% for patients with 0, 1 and 2+ features of high-risk genetics, respectively.

Despite a growing and already large body of evidence for newer effective regimens, one barrier to adopting trial regimens to routine clinical practice and even comparing data across trials is the variability of drug doses, frequency of individual drugs (subcutaneous bortezomib given on Days 1, 4, 8, and 11 of a 21 or 28-day cycle, in routine clinical practice, bortezomib is given subcutaneously on a weekly schedule with lower incidence and grade of toxicities), and cycle length (standard dose of lenalidomide given for 14 days or 21 days in a 21 or 28-day cycle) in Phase II and Phase III trials due to trial designs variations. To mitigate some of the challenges of the variability of trial designs, study populations, and dose/frequency of regimens, we have to look at the individual evidence, such as VRD-Lite data, where bortezomib was used weekly and showed comparable responses. In the Perseus trial, the dexamethasone dose was 160 mg for Weeks 1 and 2 during a 28-day cycle. Previously, a lower dose regimen with dexamethasone 40 mg weekly on Days 1, 8, 25, and 22 with lenalidomide was found to be safer, with better short-term survival and lower toxicity in a prospective Phase III trial. With these challenges, many individual oncologists and group practices may have to agree upon slight modifications in trial regimens to keep the side effects profile low without compromising the efficacy in clinical practice.

Do we still need CD38-targeting-antibody-based maintenance after CD38 targeting antibody-based induction therapy in TE NDMM patients as part of maintenance? The answer to this question is being explored in a few prospective trials. The prospective SWOGs1803 trial is a large Phase III trial with active enrolment, is looking at the question of adding daratumumab to lenalidomide post-transplant maintenance, optimal duration of maintenance, and potentially will generate data for future MRD-directed management after autologous stem cell transplantation [56]. In the Cassiopeia Phase III prospective trial, the cohort with daratumumab given as part of induction (Dara-VTD) failed to show the benefit of daratumumab after induction therapy. Phase III Perseus trial TE NDMM patients were not randomized for maintenance therapy, the cohort with daratumumab-based induction (Dara-VRD) received Dara-Len maintenance, and due to this study design limitation, this trial cannot answer the question about the role of daratumumab in the maintenance setting. Newer immunomodulatory cereblon E3 ligase modulator Iberdomide and T cell engager antibodies are being tested for maintenance therapy in TE NDMM. Despite great progress, patients on front-line therapies eventually develop drug resistance and relapse. In addition to many three-drug combinations, which are the current standard for early relapse settings, two commercial CAR T-cell therapies have shown remarkable efficacy in heavily pretreated MM patients and were recently approved for early lines of therapy. As data about these therapies progress through early line clinical trials and in NDMM, there is potential for further improvement in the OS for TE NDMM patients [57,58]. While this review highlights the constantly evolving landscape of multiple agent therapy for NDMM, summarized in Table 7, further studies will be essential to elucidating the ongoing research and validate the findings.

## 5. Conclusions

This comprehensive scoping review illuminates the dynamic landscape of treatment options for TE NDMM patients. The escalating incidence and mortality rates of multiple myeloma underscore the imperative need to refine therapeutic approaches to enhance patient outcomes. Stem cell transplantation remains a pivotal component of treatment planning for transplant-eligible patients, and triple induction therapy forms the cornerstone of initial treatment. This review reveals valuable insights into the efficacy and safety of different induction regimens, with a focus on triple and quadruple therapies.

Daratumumab, the anti-CD38 monoclonal antibody, emerges as a frequently reported and promising agent in quadruple therapy when compared to triple therapy regimens as evidenced by key trials like Perseus, CASSIOPEIA, the GRIFFIN study, and Lyra. Its favorable outcomes in induction therapy for TE NDMM patients hold potential for improved treatment responses and survival rates. In addition to daratumumab, other potential induction agents like isatuximab and carfilzomib in four-drug combination therapy demonstrate encouraging safety profiles and favorable outcomes associated with MRD and PFS.

The review also highlights the effectiveness of standard triplet therapy regimens such as VRd, VRd (lite), CyBorD, KRd, Vtd, and KTd in improving patient care and treatment outcomes in the TE NDMM patient population. This scoping review provides valuable guidance for clinicians, emphasizing the significance of individualized treatment strategies based on clinical trial evidence. By evaluating multiple induction regimens with varying efficacy and safety profiles, this review empowers clinicians to tailor treatments to meet the unique needs of different patient subsets. The findings from these trials serve as crucial benchmarks to inform future research in the quest for improved therapeutic options for newly diagnosed multiple myeloma patients eligible for stem cell transplantation.

## Figures and Tables

**Table 1:** Four-drug regimen using daratumumab.

Clinical Trail	Number of Participants and Treatment Regimen	Efficacy	Median Follow-Up (Months)
Perseus, Phase III	n = 709	48 months PFS: 84.3% vs. 67.7%	47.5
	Dara-VRd vs. VRd	CR: 87.9% vs. 70.1% MRD negative 75.5% vs. 47.5%	
Griffin, Phase II	n = 207	48 months PFS: 87.2% vs. 70%	49.6
	Dara-VRd vs. VRd	48 months CR: 83% vs. 60% 22.1 months sCR: 63% vs. 45.4% MRD negative: 64% vs. 30%	
Cassiopeia, Phase III	n = 1085	Median PFS: NR vs. 46.7 months	35.4
	Dara-VTd vs. VTd	sCR: 28.9% vs. 20.3% CR: 38.9% vs. 26%	
		VGPR: 83.4% vs. 78%	
		MRD negative: 63.7% vs. 43.5%	
Master, Phase II	n = 123 Dara-KRd	24 months PFS: 87% sCR: 84% CR: 86% VGPR: 98% MRD negative: 80%	25.1
Lyra, Phase II	n = 87 Dara-Vcd	Median PFS: NR 36 months PFS: 69.3% 1 year PFS 87% sCR: 23.1% CR: 49% VGPR: 82%	35.7

CR—complete response; Dara—daratumumab; KRd—carfilzomib, lenalidomide, dexamethasone; MRD—minimal residual disease; NR—no response; PFS—progression-free survival; sCR—stringent complete response; VCd—bortezomib, cyclophosphamide, and dexamethasone; VGPR—very good partial response; VRd—bortezomib, lenalidomide, and dexamethasone; VTd—bortezomib, thalidomide, and dexamethasone.

**Table 2:** Toxicity of myeloma regimen using daratumumab.

Clinical Trial	Treatment Regimen	Most Common Adverse Events: Grade 3/4
Perseus, Phase III	Dara-VRd vs. VRd	Neutropenia 62.1% vs. 51 Thrombocytopenia 29.1% vs. 17.3% Diarrhea 10.5% vs. 7.8%
Griffin, Phase II	Dara-VRd vs. VRd	Neutropenia 37.0% vs. 29.6% Lymphopenia 25.9% vs. 11.1%
Cassiopeia, Phase III	Dara-VTd vs. VTd	Neutropenia 27.6% vs. 14.7% Lymphopenia 17% vs. 9.7% Stomatitis 12.7% vs. 16.4% Thrombocytopenia 11% vs. 7.4%
Master, Phase II	Dara-KRd	Neutropenia 35% Lymphopenia 22% Anemia 11% Hypertension 10% Fatigue 9%
Lyra, Phase II	Dara-Vcd	Neutropenia 12.8% Fatigue 7% Leukopenia 5.8% Diarrhea 4.7% Pneumonia 3.5%

Dara—daratumumab; KRd—carfilzomib, lenalidomide, dexamethasone; VCd—bortezomib, cyclophosphamide, and dexamethasone; VRd—bortezomib, lenalidomide, and dexamethasone; VTd—bortezomib, thalidomide, and dexamethasone.

**Table 3:** Four-drug regimen using isatuximab, carfilzomib, elotuzumab, and cyclophosphamide.

Clinical Trail	Number of Participants and Treatment Regimen	Efficacy	Median Follow-Up (Months)
GMMG-HD7, Phase III	n = 660	CR: 24% vs. 22%	4.1
	Isa-VRd vs. VRd	nCR: 41% vs. 37% VGPR: 77% vs. 61% PR or better: 90% vs. 84% MRD negative: 50% vs. 36%	
Myeloma XI+, Phase III	n = 1056 KRdc vs. Rdc/Tdc	At 100 days post ASCT: CR: 31% vs. 24% nCR: 38.6% vs. 31.7% VGPR: 22.3% vs. 23.7% MRD negative: 75.2% vs. 50%Median PFS: NR vs. 36.2 months 3 years PFS: 64.5% vs. 50.3%	34.5
SWOG-1211, Phase II	n = 100 Elo-VRd vs. VRd	CR: 2.1% vs. 6% VGPR: 21.3% vs. 20% PR: 59.6% vs. 62% ORR: 83% vs. 88% Median PFS: 31.47 vs. 33.64 months	53
Evolution, Phase II	n = 140 VdcR-, VRd-, Vdc-, and Vdc-modified	CR: 25% vs. 10% vs. 22% vs. 47% sCR: 15% vs. 17% vs. 9% vs. 29% VGPR: 58% vs. 51% vs. 41% vs. 53% ORR: 88% vs. 85% vs. 75% vs. 100%	20

ASCT—autologous stem cell transplant; CR—complete response; Elo—elotuzumab; Isa—isatuximab; KRDc—carfilzomib, lenalidomide, dexamethasone, and cyclophosphamide; MRD—minimal residual disease; nCR—near-complete response; ORR—objective response rate; PFS—progression-free survival; PR—partial response; Rdc—lenalidomide, dexamethasone, and cyclophosphamide; sCR—stringent complete response; Tdc—thalidomide, dexamethasone, and cyclophosphamide; Vdc—bortezomib, dexamethasone, and cyclophosphamide; VdcR—bortezomib, dexamethasone, cyclophosphamide, lenalidomide; VGPR—very good partial response; VRd—bortezomib, lenalidomide, and dexamethasone.

**Table 4:** Toxicity of myeloma regimen using isatuximab, carfilzomib, elotuzumab, and cyclophosphamide.

Clinical Trial	Treatment Regimen	Most Common Adverse Events: Grade 3/4
GMMG-HD7, Phase III	Isa-VRd vs. VRd	Blood and lymphatic system disorder 26% vs. 17% Neutropenia 23% vs. 7% Lymphopenia 15% vs. 20% Infection 12% vs. 10%
Myeloma XI+, Phase III	KRdc vs. Rdc/Tdc	Neutropenia 16.4% vs. 22.2%/12.4% Infections 16.1% vs. 11.5%/11.7% Anemia 10.2% vs. 5.8%/4.7%
SWOG-1211, Phase II	Elo-VRd vs. VRd	Nervous system disorder 21% vs. 12% Blood and lymphatic 15% vs. 17% Infections 17% vs. 8% Vascular disorders 13% vs. 16%
Evolution, Phase II	VdcR, VRd, Vdc and Vdc-mod	Neutropenia 44% vs. 10% vs. 30% vs. 24% Neuropathy 13% vs. 17% vs. 9% vs. 18% Fatigue 17% vs. 7% vs. 3% vs. 0%

ASCT—autologous stem cell transplant; CR—complete response; Elo—elotuzumab; Isa—isatuximab; KRDc—carfilzomib, lenalidomide, dexamethasone, and cyclophosphamide; MRD—minimal residual disease; nCR—near-complete response; ORR—objective response rate; PFS—progression-free survival; PR—partial response; Rdc—lenalidomide, dexamethasone, and cyclophosphamide; sCR—stringent complete response; Tdc—thalidomide, dexamethasone, and cyclophosphamide; Vdc—bortezomib, dexamethasone, and cyclophosphamide; VdcR—bortezomib, dexamethasone, cyclophosphamide, lenalidomide; VGPR—very good partial response; VRd—bortezomib, lenalidomide, and dexamethasone.

**Table 5:** Three Drug Regimen.

Clinical Trail	Number of Participants & Treatment Regimen	Efficacy	Median Follow-Up (Months)
PETHEMA/GEM2012	n = 458 VTd	PFS: NR CR post-induction: 33.4% CR post-ASCT: 44.1% CR post-consolidation: 50% MRD positive post-induction: 57.6% MRD positive post-ASCT: 36.5% MRD positive post-consolidation: 34.3%	24.2
Endurance	n = 1087	PFS median: 34.4 vs. 34.6 months	9.4
	VRd vs. KRd	ORR: 84% vs. 87% VGPR 65% vs. 74% PR 84% vs. 87% CR 15% vs. 18% MRD-negative 7% vs. 10%	
Reeder Trial	n = 63 Vcd vs. modified Vcd	Overall PFS: 42% ORR: 88% vs. 93% VGPR: 61% vs. 60% CR/nCR: 39% vs. 40%	12.4
VRd Lite [37]	n = 48 Modified VRd	ORR: 83% ORR Post ASCT: 100% VGPR Post ASCT: 74% CR: 25%	NA
NCT02405364	n = 46 KRd	Median PFS: 56.4 months VGPR: 92.9% ORR: 97.7% sCR: 56.5% CR post-induction: 23% CR post-ASCT: 41.5% CR post-consolidation: 64.5%	60.5
Gimema-MMY-3006	n = 480 VTd vs. Td	Median PFS: 60 vs. 41 months 10 years PFS: 34% vs. 17% CR: 27% vs. 15% OS at 10 years: 60% vs. 46%	124.1
Carthadex	n = 111 KTd	Median PFS: 58 months ORR: 90% VGPR: 68% Median OS: 83 months 5-year OS: 76% CR post-induction: 18% CR post-melphalan: 31% CR post-consolidation: 63%	58.7
FORTE	n = 477 KRd vs. Kcd	sCR: 46% vs. 32% MRD negativity: 62% vs. 43% 4-year PFS: 69% vs. 51% 4-year OS: 86% vs. 76%	50.9
Determination	n = 722 VRd vs. VRd+ASCT	Median PFS: 46.2 vs. 67.6 months CR: 52 vs. 62% VGPR: 79% vs. 83% PR: 94% vs. 96% MRD negativity: 37.3% vs. 52.1% 4 years OS: 84% vs. 85%	76
IFM 2009	n = 70 VRd vs. VRd+ASCT	CR: 48% vs. 59% VGPR: 29% vs. 29% PR: 20% vs. 11% MRD: 65% vs. 79%	44
SWOG S0777	n = 525 VRd vs. Rd	Median PFS: 43 vs. 30 months ORR: 82% vs. 72% VGPR: 27.8% vs. 23.4% CR: 16% vs. 8% Median OS: 75 vs. 64 months	55

ASCT—autologous stem cell transplant; CR—complete response; Kcd—carfilzomib, cyclophosphamide, dexamethasone; KRd—carfilzomib, lenalidomide, dexamethasone; KTd—carfilzomib, thalidomide, dexamethasone; MRD—minimal residual disease; nCR—near-complete response; ORR—objective response rate; OS—overall survival; PFS—progression-free survival; PR—partial response; Rd—lenalidomide, and dexamethasone; Td—thalidomide and dexamethasone; VGPR—very good partial response; VRd—bortezomib, lenalidomide, and dexamethasone; VTd—dortezomib, thalidomide, and dexamethasone; Vdc—bortezomib, dexamethasone, cyclophosphamide.

**Table 6:** Toxicity of myeloma three-drug regimen.

Clinical Trial	Treatment Regimen	Most Common Adverse Events: Grade 3/4
PETHEMA/GEM2012	VRd	Neutropenia 12.9% Infection 9.2% Thrombocytopenia 6.3%
Endurance	VRd vs. KRd	Peripheral neuropathy 8% vs. <1% Fatigue 6% vs. 6% Hyperglycemia 4% vs. 6% Diarrhea 5% vs. 3%
VRd Lite	Modified VRd	Neutropenia 19%, Lymphocytopenia 8% Infection 6%
NCT02405364	KRd	Lymphopenia 65.2% Neutropenia 34.8% Thrombocytopenia 19.6%
Gimema-MMY-3006	VTd vs. Td	n/a
Carthadex	KTd	Hematologic toxicity 10% Infection 11% Dermatologic 9% Vascular disorders 9% Respiratory disorder 8%
FORTE	KRd vs. Kcd	Neutropenia: 13% vs. 11% Dermatological toxicity: 6% vs. 1% Hepatic toxicity: 8% vs. 0%
Determination	VRd+ASCT vs. VRd	Any hematologic event: 60.5% vs. 89.9% Gastrointestinal disorder: 7.8% vs. 18.6% Infections: 9.5% vs. 18.4%
IFM 2009	VRd vs. VRd+ASCT	Any hematologic event: 63.7% vs. 94.9% Infections: 8.9% vs. 20.3% Gastrointestinal disorder: 6.9% vs. 27.7%
SWOG S0777	VRd vs. Rd	Anemia: 13% vs. 16% Fatigue: 16% vs. 14% Neuropathy: 23% vs. 3%

ASCT—autologous stem cell transplant; Kcd—carfilzomib, cyclophosphamide, dexamethasone; KRd—carfilzomib, lenalidomide, dexamethasone; KTd—carfilzomib, thalidomide, dexamethasone; Rd—lenalidomide, and dexamethasone; Td—thalidomide and dexamethasone; VRd—bortezomib, lenalidomide, and dexamethasone; VTd—bortezomib, thalidomide, and dexamethasone. n/a: not reported.

**Table 7:** Ongoing clinical trials for NDMM.

Ongoing Clinical Trial	Treatment Regimen	Purpose of the Trial
EUCTR2018-002089-37-GR 2018 [59]	Dara-Vcd vs. VTd	PFS at 36 months
EUCTR2018-002992-16-GR 2018 [60]	Dara-VRd vs. VRd	PFS
EUCTR2019-004844-32-GR 2020(IsKia Trial) [59]	Isa-KRd vs. KRd	MRD negativity
NCT03896737 2019 [61]	Dara-Vcd vs. VTd	PFS

Dara—daratumumab; Isa—isatuximab; KRd—carfilzomib, lenalidomide, dexamethasone; MRD—minimal residual disease; PFS—progression-free survival; VCd—bortezomib, cyclophosphamide, and dexamethasone; VRd—bortezomib, lenalidomide, and dexamethasone; VTd—bortezomib, thalidomide, and dexamethasone.

## Data Availability

No new data were created or analyzed in this study. Data sharing is not applicable to this article.

## References

[1] American Cancer Society Cancer Statistics Center. Published March 31, 2024. https://cancerstatisticscenter.cancer.org/#!/.

[2] International Myeloma Working Group International Myeloma Working Group (IMWG) Criteria for the Diagnosis of Multiple Myeloma. International Myeloma Foundation. https://www.myeloma.org/international-myeloma-working-group-imwg-criteria-diagnosis-multiple-myeloma.

[3] In Proceedings of the 23rd Congress of the European Hematology Association Stockholm, Sweden, 14–17 June 2018; HemaSphere: Stockholm, Sweden, 2018; Volume 2 (Suppl. S1), pp. 1–1113. https://www.ncbi.nlm.nih.gov/pmc/articles/PMC6110645/.

[4] In Proceedings of the 46th Annual Meeting of the European Society for Blood and Marrow Transplantation: Physicians Oral Session (O010-O173); Bone Marrow Transplantation: Baltimore, MD, USA, 2020; Volume 55 (Suppl. S1), pp. 22–174. https://www.nature.com/articles/s41409-020-01118-4.

[5] D’Agostino M., Cairns D.A., Lahuerta J.J., Wester R., Bertsch U., Waage A., Zamagni E., Mateos M.-V., Dall’Olio D., van de Donk N.W.C.J. (2022). Second revision of the International Staging System (R2-ISS) for overall survival in multiple myeloma: A European Myeloma Network (EMN) report within the HARMONY project. J. Clin. Oncol..

[6] National Comprehensive Cancer Network NCCN Clinical Practice Guidelines in Oncology. https://www.nccn.org/guidelines/guidelines-detail?category=1&id=1445.

[7] Rajkumar S.V. (2016). Updated Diagnostic Criteria and Staging System for Multiple Myeloma. Am. Soc. Clin. Oncol. Educ. Book.

[8] Rajkumar S.V. (2022). Multiple myeloma: 2022 update on diagnosis, risk stratification, and management. Am. J. Hematol..

[9] Rajkumar S.V., Dimopoulos M.A., Palumbo A., Blade J., Merlini G., Mateos M.-V., Kumar S., Hillengass J., Kastritis E., Richardson P. (2014). International Myeloma Working Group updated criteria for the diagnosis of multiple myeloma. Lancet Oncol..

[10] Cowan A.J., Green D.J., Kwok M., Lee S., Coffey D.G., Holmberg L.A., Tuazon S., Gopal A.K., Libby E.N. (2022). Diagnosis and Management of Multiple Myeloma: A Review. JAMA.

[11] Boussi L.S., Avigan Z.M., Rosenblatt J. (2022). Immunotherapy for the treatment of multiple myeloma. Front. Immunol..

[12] Kegyes D., Constantinescu C., Vrancken L., Rasche L., Gregoire C., Tigu B., Gulei D., Dima D., Tanase A., Einsele H. (2022). Patient selection for CAR T or BiTE therapy in multiple myeloma: Which treatment for each patient?. J. Hematol. Oncol..

[13] Rejeski K., Jain M.D., Smith E.L. (2023). Mechanisms of Resistance and Treatment of Relapse after CAR T-cell Therapy for Large B-cell Lymphoma and Multiple Myeloma. Transplant Cell Ther..

[14] International Myeloma Foundation Multiple Myeloma Drugs. International Myeloma Foundation. https://www.myeloma.org/multiple-myeloma-drugs.

[15] Sonneveld P., Dimopoulos M.A., Boccadoro M., Quach H., Ho P.J., Beksaç M., Hulin C., Antonioli E., Leleu X., Mangiacavalli S. (2013). Daratumumab, bortezomib, lenalidomide, and dexamethasone for multiple myeloma. N. Engl. J. Med..

[16] Sonneveld P., Dimopoulos M.A., Boccadoro M., Quach H., Ho P.J., Beksaç M., Hulin C., Antonioli E., Leleu X., Mangiacavalli S. (2013). Phase 3 Randomized Study of Daratumumab (DARA) + Bortezomib, Lenalidomide, and Dexamethasone (VRd) Versus Vrd Alone in Patients (Pts) with Newly Diagnosed Multiple Myeloma (NDMM) Who Are Eligible for Autologous Stem Cell Transplantation (ASCT): Primary Results of the Perseus Trial. Blood.

[17] Sborov D.W., Baljevic M., Reeves B., Laubach J., Efebera Y.A., Rodriguez C., Costa L.J., Chari A., Silbermann R., Holstein S.A. (2022). Daratumumab plus lenalidomide, bortezomib and dexamethasone in newly diagnosed multiple myeloma: Analysis of vascular thrombotic events in the GRIFFIN study. Br. J. Haematol..

[18] Voorhees P.M., Kaufman J.L., Laubach J.P., Sborov D.W., Reeves B., Rodriguez C., Chari A., Silbermann R., Costa L.J., Anderson L.D. (2022). Daratumumab, Lenalidomide, Bortezomib, & Dexamethasone for Transplant-eligible Newly Diagnosed Multiple Myeloma: GRIFFIN. Blood J. Am. Soc. Hematol..

[19] Voorhees P.M., Kaufman J.L., Laubach J., Sborov D.W., Reeves B., Rodriguez C., Chari A., Silbermann R., Costa L.J., Anderson L.D. (2022). Depth of Response to Daratumumab, Lenalidomide, Bortezomib, and Dexamethasone Improves over Time in Patients with Transplant-Eligible Newly Diagnosed Multiple Myeloma: Griffin Study Update.

[20] Voorhees P.M., Rodriguez C., Reeves B., Nathwani N., Costa L.J., Lutska Y., Bobba P., Hoehn D., Pei H., Ukropec J. (2021). Daratumumab plus VRd for newly diagnosed multiple myeloma: Final analysis of the safety run-in cohort of GRIFFIN. Blood Adv..

[21] Chari A., Kaufman J.L., Laubach J., Sborov D.W., Reeves B., Rodriguez C., Silbermann R., Costa L.J., Anderson L.D., Nathwani N. (2024). Daratumumab in transplant-eligible patients with newly diagnosed multiple myeloma: Final analysis of clinically relevant subgroups in GRIFFIN. Blood Cancer J..

[22] Touzeau C., Moreau P., Perrot A., Hulin C., Dib M., Tiab M., Caillot D., Facon T., Leleu X., van de Donk N.W.C.J. (2020). Daratumumab + bortezomib, thalidomide, and dexamethasone (D-VTd) in transplant-eligible newly diagnosed multiple myeloma (TE NDMM): Baseline SLiM-CRAB based subgroup analysis of CASSIOPEIA. J. Clin. Oncol..

[23] Moreau P., Sonneveld P. (2021). Daratumumab (DARA) maintenance or observation (OBS) after treatment with bortezomib, thalidomide and dexamethasone (VTd) with or without DARA and autologous stem cell transplant (ASCT) in patients (pts) with newly diagnosed multiple myeloma (NDMM): CASSIOPEIA Part 2. J. Clin. Oncol..

[24] Sonneveld P., Attal M., Perrot A., Hulin C., Caillot D., Facon T., Leleu X., Belhadj-Merzoug K., Karlin L., Benboubker L. (2019). Daratumumab plus bortezomib, thalidomide, and dexamethasone (D-VTD) in transplant-eligible Newly Diagnosed Multiple myeloma (NDMM): Subgroup Analysis of High-risk Patients (PTS) in CASSIOPEIA. Clin. Lymphoma Myeloma Leuk..

[25] Costa L.J., Chhabra S., Medvedova E., Dholaria B.R., Schmidt T.M., Godby K.N., Silbermann R., Dhakal B., Bal S., Giri S. (2022). Daratumumab, Carfilzomib, Lenalidomide, and Dexamethasone with Minimal Residual Disease Response-Adapted Therapy in Newly Diagnosed Multiple Myeloma. J. Clin. Oncol..

[26] Rifkin R., Melear J., Faber E., Bensinger W., Burke J., Narang M., Stevens D., Gray K., Lutska Y., Bobba P. (2021). Daratumumab plus cyclophosphamide, bortezomib, and dexamethasone induction therapy in multiple myeloma followed by daratumumab maintenance: End-of-study results from lyra. HemaSphere.

[27] Yimer H., Melear J., Faber E., Bensinger W.I., Burke J.M., Narang M., Stevens D., Gunawardena S., Lutska Y., Qi K. (2019). Daratumumab, bortezomib, cyclophosphamide and dexamethasone in newly diagnosed and relapsed multiple myeloma: LYRA study. Br. J. Haematol..

[28] Yimer H., Melear J., Faber E., Bensinger W.I., Burke J.M., Narang M., Stevens D., Gray K.S., Lutska Y., Bobba P. (2022). Daratumumab, cyclophosphamide, bortezomib, and dexamethasone for multiple myeloma: Final results of the LYRA study. Leuk. Lymphoma.

[29] Goldschmidt H., Mai E.K., Bertsch U., Fenk R., Nievergall E., Tichy D., Besemer B., Dürig J., Schroers R., von Metzler I. (2022). Addition of isatuximab to lenalidomide, bortezomib, and dexamethasone as induction therapy for newly diagnosed, transplantation-eligible patients with multiple myeloma (GMMG-HD7): Part 1 of an open-label, multicentre, randomised, active-controlled, phase 3 trial. Lancet Haematol..

[30] Subgroup Analysis of Phase 3 Trial GMMG-HD7 Evaluating Isa-VRd in Patients with High-Risk Cytogenetics. Oncology Practice Management. https://oncpracticemanagement.com/web-exclusive-articles/2883:subgroup-analysis-of-phase-3-trial-gmmg-hd7-evaluating-isa-VRd-in-patients-with-high-risk-cytogenetics.

[31] Goldschmidt H., Mai E.K., Nievergall E., Fenk R., Bertsch U., Tichy D., Besemer B., Dürig J., Schroers R., Metzler I.V. (2021). Addition of isatuximab to lenalidomide, bortezomib, and dexamethasone as induction therapy for newly diagnosed, transplant-eligible multiple myeloma patients: The phase III GMMG-HD7 trial. Blood.

[32] Mai E.K., Bertsch U., Fenk R., Tichy D., Besemer B., Dürig J., Schroers R., von Metzler I., Hänel M., Mann C. Isatuximab, lenalidomide, bortezomib, and dexamethasone as induction therapy for newly diagnosed multiple myeloma patients with high-risk cytogenetics: A subgroup analysis from the GMMG-HD7 trial. Proceedings of the European Hematology Association Congress.

[33] Jackson G.H., Pawlyn C., Cairns D.A., de Tute R.M., Hockaday A., Collett C., Jones J.R., Kishore B., Garg M., Williams C.D. (2021). Carfilzomib, lenalidomide, dexamethasone, and cyclophosphamide (KRdc) as induction therapy for transplant-eligible, newly diagnosed multiple myeloma patients (Myeloma XI+): Interim analysis of an open-label randomised controlled trial. PLoS Med..

[34] Panopoulou A., Cairns D.A., Holroyd A., Nichols I., Cray N., Pawlyn C., Cook G., Drayson M., Boyd K., Davies F.E. (2023). Optimizing the value of lenalidomide mainten+ance by extended genetic profiling: An analysis of 556 patients in the Myeloma XI trial. Blood.

[35] Usmani S.Z., Hoering A., Ailawadhi S., Sexton R., Lipe B., Hita S.F., Valent J., Rosenzweig M., Zonder J.A., Dhodapkar M. (2021). Bortezomib, lenalidomide, and dexamethasone with or without elotuzumab in patients with untreated, high-risk multiple myeloma (SWOG-1211): Primary analysis of a randomised, phase 2 trial. Lancet Haematol..

[36] Kumar S., Flinn I., Richardson P.G., Hari P., Callander N., Noga S.J., Stewart A.K., Turturro F., Rifkin R., Wolf J. (2012). Randomized, multicenter, phase 2 study (EVOLUTION) of combinations of bortezomib, dexamethasone, cyclophosphamide, and lenalidomide in previously untreated multiple myeloma. Blood.

[37] Okazuka K., Ishida T., Nashimoto J., Uto Y., Sato K., Miyazaki K., Ogura M., Yoshiki Y., Abe Y., Tsukada N. (2019). The efficacy and safety of modified bortezomib-lenalidomide-dexamethasone in transplant-eligible patients with newly diagnosed multiple myeloma. Eur. J. Haematol..

[38] Rosiñol L., Oriol A., Ríos R., Sureda A., Blanchard M.J., Hernández M.T., Martínez-Martínez R., Moraleda J.M., Jarque I., Bargay J. (2019). Bortezomib, lenalidomide, and dexamethasone as induction therapy prior to autologous transplant in multiple myeloma. Blood.

[39] Kumar S.K., Jacobus S.J., Cohen A.D., Weiss M., Callander N., Singh A.K., Parker T.L., Menter A., Yang X., Parsons B. (2020). Carfilzomib or bortezomib in combination with lenalidomide and dexamethasone for patients with newly diagnosed multiple myeloma without intention for immediate autologous stem-cell transplantation (ENDURANCE): A multicentre, open-label, phase 3, randomised, controlled trial. Lancet Oncol..

[40] Reeder C.B., Reece D.E., Kukreti V., Mikhael J., Chen C.I., Trudel S., Laumann K., Hentz J., Piza G., Fonseca R. (2013). Long-term survival with CYBORD induction therapy in newly diagnosed multiple myeloma. Blood.

[41] Reeder C.B., Reece D.E., Kukreti V., Mikhael J.R., Chen C., Trudel S., Laumann K., Hentz J., Pirooz N., Piza J. (2009). A phase II trial comparison of once versus twice weekly bortezomib in CYBORD chemotherapy for newly diagnosed myeloma: Identical high response rates and less toxicity. Blood.

[42] Muranushi H., Kanda J., Kobayashi M., Maeda T., Kitano T., Tsuji M., Ueda Y., Ishikawa T., Nohgawa M., Watanabe M. (2022). Bortezomib-cyclophosphamide-dexamethasone induction/consolidation and bortezomib maintenance for transplant-eligible newly diagnosed multiple myeloma: Phase 2 multicenter trial. Hematology.

[43] Roussel M., Lauwers-Cancès V., Wuillème S., Belhadj K., Manier S., Garderet L., Escoffre-Barbe M., Mariette C., Benboubker L., Caillot D. (2021). Up-front carfilzomib, lenalidomide, and dexamethasone with transplant for patients with multiple myeloma: The IFM KRd final results. Blood.

[44] Gay F., Musto P., Rota-Scalabrini D., Bertamini L., Belotti A., Galli M., Offidani M., Zamagni E., Ledda A., Grasso M. (2021). Carfilzomib with cyclophosphamide and dexamethasone or lenalidomide and dexamethasone plus autologous transplantation or carfilzomib plus lenalidomide and dexamethasone, followed by maintenance with carfilzomib plus lenalidomide or lenalidomide alone for patients with newly diagnosed multiple myeloma (FORTE): A randomized, open-label, phase 2 trial. Lancet Oncol..

[45] Mina R., Musto P., Rota-Scalabrini D., Paris L., Gamberi B., Palmas A., Aquino S., Paolo de Fabritiis Giuliani N., Luca De Rosa Alessandro Gozzetti Cellini C., Luca Bertamini Capra A. (2022). Carfilzomib induction, consolidation, and maintenance with or without autologous stem-cell transplantation in patients with newly diagnosed multiple myeloma: Pre-planned cytogenetic subgroup analysis of the randomised, phase 2 FORTE trial. Lancet Oncol..

[46] ClinicalTrials.gov. https://clinicaltrials.gov/study/NCT01816971.

[47] Richardson P.G., Jacobus S.J., Weller E., Hassoun H., Lonial S., Raje N.S., Medvedova E., McCarthy P.L., Libby E.N., Voorhees P. (2022). Lenalidomide, bortezomib, and dexamethasone (VRd) ± autologous stem cell transplantation (ASCT) and R maintenance to progression for newly diagnosed multiple myeloma (NDMM): The phase 3 DETERMINATION trial. J. Clin. Oncol..

[48] Richardson P.G., Jacobus S.J., Weller E.A., Hassoun H., Lonial S., Raje N.S., Medvedova E., McCarthy P.L., Libby E.N., Voorhees P.M. (2022). Triplet Therapy, Transplantation, and Maintenance until Progression in Myeloma. N. Engl. J. Med..

[49] Attal M., Lauwers-Cances V., Hulin C., Leleu X., Caillot D., Escoffre M., Arnulf B., Macro M., Belhadj K., Garderet L. (2017). Lenalidomide, Bortezomib, and Dexamethasone with Transplantation for Myeloma. N. Engl. J. Med..

[50] Durie B.G.M., Hoering A., Abidi M.H., Rajkumar S.V., Epstein J., Kahanic S.P., Thakuri M., Reu F., Reynolds C.M., Sexton R. (2017). Bortezomib with lenalidomide and dexamethasone versus lenalidomide and dexamethasone alone in patients with newly diagnosed myeloma without intent for immediate autologous stem-cell transplant (SWOG S0777): A randomised, open-label, phase 3 trial. Lancet.

[51] Tacchetti P., Pantani L., Patriarca F., Petrucci M.T., Zamagni E., Dozza L., Galli M., Di Raimondo F., Crippa C., Boccadoro M. (2020). Bortezomib, thalidomide, and dexamethasone followed by double autologous haematopoietic stem-cell transplantation for newly diagnosed multiple myeloma (GIMEMA-MMY-3006): Long-term follow-up analysis of a randomised phase 3, open-label study. Lancet Haematol..

[52] Tacchetti P., Patriarca F., Petrucci M.T., Galli M., Pantani L., Dozza L., Di Raimondo F., Boccadoro M., Offidani M., Montefusco V. (2018). A triplet bortezomib-and immunomodulator-based therapy before and after double ASCT improves overall survival of newly diagnosed mm patients: Final analysis of phase 3 gimema-MMY-3006 study. HemaSphere.

[53] Wester R., van der Holt B., Asselbergs E., Zweegman S., Kersten M.J., Vellenga E., Kooy M.v.M., de Weerdt O., Minnema M., Lonergan S. (2019). Phase II study of carfilzomib, thalidomide, and low-dose dexamethasone as induction and consolidation in newly diagnosed, transplant eligible patients with multiple myeloma; the Carthadex trial. Haematologica.

[54] Gordan L.N., Marks S.M., Xue M., Nagovski N., Lambert J.H., Smith R.E. (2022). Daratumumab utilization and cost analysis among patients with multiple myeloma in a US community oncology setting. Future Oncol..

[55] (2024). Meeting of the Oncologic Drugs Advisory Committee Meeting Announcement—04/12/2024. FDA. https://www.fda.gov/advisory-committees/advisory-committee-calendar/april-12-2024-meeting-oncologic-drugs-advisory-committee-meeting-announcement-04122024.

[56] National Cancer Institute (2016). Clinical Trials Search. https://www.cancer.gov/research/participate/clinical-trials-search/v?loc=0&tid=S1803&rl=2&id=NCI-2018-02465&pn=1&ni=10.

[57] Manier S., Ingegnere T., Escure G., Prodhomme C., Nudel M., Mitra S., Facon T. (2022). Current state and next-generation CAR-T cells in multiple myeloma. Blood Rev..

[58] Yakoub-Agha I., Einsele H., Kröger N., Gribben J., Chabannon C., Yakoub-Agha I., Einsele H. (2022). Multiple myeloma. The EBMT/EHA CAR-T Cell Handbook.

[59] EUCTR GR A Multicenter, Open-Label, Randomized Phase II Study Comparing Daratumumab Combined with Bortezomib-Cyclophosphamide-Dexamethasone (DARA-VCD) versus the Association of Bortezomib-Thalidomide-Dexamethasone (VTD) as Pre-Transplant Induction and Post-Transplant Consolidation, both followed by a Maintenance Phase with Ixazomib Alone or in Combination with Daratumumab, in Newly Diagnosed Multiple Myeloma (MM) Young Patients Eligible for Autologous Stem Cell Transplantation. https://trialsearch.who.int/trial2.aspx?trialid=EUCTR2018-002089-37-GR.

[60] EUCTR GR A Study of Combination of Daratumumab, VELCADE (Bortezomib), Lenalidomide, and Dexamethasone (D-VRd) Compared to VELCADE, Lenalidomide, and Dexamethasone (VRd) in Participants with Previously Untreated Multiple Myeloma. https://www.clinicaltrials.gov/study/NCT03652064.

[61] Daratumumab-Bortezomib-Dexamethasone (Dara-VCD) vs. Bortezomib-Thalidomide-Dexamethasone (VTd) then Maintenance with Ixazomib (IXA) or IXA-Dara—Full Text View. https://classic.clinicaltrials.gov/ct2/show/NCT03896737.

[62] Lytvynova O., Jwayyed J., Hameed M., Baloch A., Prasad R., Salman A., Rafae A., Mukhopadhyay D., Kumarasamy V., Answer F. (2023). Evidence-Based Recommendations for Induction Treatment of Newly Diagnosed Transplant-Eligible Multiple Myeloma Patients; A Scoping Review. Blood.

